# Inferring phenomenological models of first passage processes

**DOI:** 10.1371/journal.pcbi.1008740

**Published:** 2021-03-05

**Authors:** Catalina Rivera, David Hofmann, Ilya Nemenman

**Affiliations:** 1 Department of Physics, Emory University, Atlanta, Georgia, United States of America; 2 Initiative in Theory and Modeling of Living Systems, Emory University, Atlanta, Georgia, United States of America; 3 Department of Biology, Emory University, Atlanta, Georgia, United States of America; University of Pittsburgh, UNITED STATES

## Abstract

Biochemical processes in cells are governed by complex networks of many chemical species interacting stochastically in diverse ways and on different time scales. Constructing microscopically accurate models of such networks is often infeasible. Instead, here we propose a systematic framework for building *phenomenological* models of such networks from experimental data, focusing on accurately approximating the time it takes to complete the process, the First Passage (FP) time. Our phenomenological models are mixtures of Gamma distributions, which have a natural biophysical interpretation. The complexity of the models is adapted automatically to account for the amount of available data and its temporal resolution. The framework can be used for predicting behavior of FP systems under varying external conditions. To demonstrate the utility of the approach, we build models for the distribution of inter-spike intervals of a morphologically complex neuron, a Purkinje cell, from experimental and simulated data. We demonstrate that the developed models can not only fit the data, but also make nontrivial predictions. We demonstrate that our coarse-grained models provide constraints on more mechanistically accurate models of the involved phenomena.

## Author summary

Building microscopically accurate models of biological processes that offer meaningful information about the behavior of these systems is a hard task that requires a lot of prior knowledge and experimental data that are not available most of the time. Here instead we propose a mathematical framework to infer phenomenological models of biochemical systems, focusing on approximating the probability distribution of the time it takes to complete the process. We apply the method to study statistical properties of spiking in morphologically complex neurons, Purkinje cells, and make nontrivial predictions about this system.

## Introduction

Processes in living cells are governed by complex networks of stochastically interacting biochemical species. Understanding such processes holistically does not necessarily imply having a detailed description of the system at a microscopic, mechanistic level. Indeed, many microscopic networks can result in equivalent experimentally observable behaviors [1], so that distinguishing alternative networks may be impossible. Even if competing models are not exactly equivalent, they may approximate each other in many key measurable behaviors [2]. Thus a lot of ink has been expended on developing methods for constructing reduced, coarse-grained models of biological processes as alternatives to unidentifiable mechanistically accurate ones [3–17]. This is usually a challenging task, requiring construction of a (possibly inaccurate) detailed mechanical model as an intermediate step. In this paper, we focus on an alternative approach of *refining* phenomenological models of stochastic biological processes rather than coarse-graining mechanistic ones. Our approach optimally adapts the level of complexity to match the amount and quality of the experimental observations while accurately predicting specific macroscopic properties of the processes.

A large number of biological processes—and the sole focus of this work—can be viewed as First Passage (FP), or completion processes [18–22]: certain molecules must interact, certain compounds must be created, or certain states must be visited, before an event of interest occurs. For such systems, one is often interested in when the final event occurs (i.e., what the FP time is), rather than in details of which molecules got created or which states were visited in the process. Thus such systems represent a fruitful field for coarse-grained modeling. Crucially, often the available experimental data are sufficiently precise to allow investigation of the whole probability distribution of the FP time, and the fact that the time is stochastic and often broadly distributed can have important functional effects [19, 23–25].

A natural approach to characterizing the FP distribution based strictly on the statistical information contained in the samples of the FP time involves progressively estimating its higher order cumulants. However, this approach suffers from a well-known problem that such cumulant expansions cannot be truncated at any order but the second, and still give rise to a proper probability distribution [26]. Here we propose a different method for systematically inferring phenomenological models of first passage distributions from empirical data. The approach does not strive for the mechanistic accuracy. Instead, following ideas from [27], we develop a family of models of FP processes, whose complexity can be grown adaptively as data requires, to fit arbitrary FP time distributions. We then choose the optimal model of the appropriate complexity within the family using Bayesian model selection [28–33].

Our model family consists of mixtures of Gamma distributions. As we will show, in the context of the FP kinetics, this represents models with multiple independent paths from the start to completion. In the well-sampled regime, this representation allows us to infer mechanistic constraints on the underlying kinetics using fits within our model family [34]. Specifically, the element of the mixture that dominates the passage for short times, sets the minimal number of internal states that a mechanistically accurate stochastic process would need to generate the data. Furthermore, our approach provides a framework to study effects of external perturbations or experimental conditions on the first passage statistics in a systematic way. Specifically, by doing model selection simultaneously on all data sets across multiple experimental conditions, we can obtain a single phenomenological model that explains all of the available data, relating parameters of such global model to the values characterizing the perturbations. Notice that Bayesian parameter inference and model selection has been applied multiple times in computational biology [35–38], however it has not been applied previously to phenomenological inference for FP processes, and especially across multiple data sets.

We test the utility of our approach on neurophysiological data sets. Most neurons are too complex to be modeled mechanistically with molecular accuracy, so that any model will involve some element of phenomenology, making this a good testing ground for our approach. Indeed, spontaneous activity of neurons of different types is often modeled under the assumption that the spike trains can be described by renewal processes [39–45]. An important characterization of such spike trains is the distribution of times between successive spikes, also known as the inter-spike intervals (ISIs). In such renewal process models, all ISIs are independent and identically distributed, and the spike generation can be specified fully by the ISI distribution, which, in turn, can be seen as produced by a FP process. It has long been understood that changes of the ISI distribution are biologically interesting since they are informative of changes in biophysical states of neurons or neural networks they form [46]. For instance, whether a network is in a normal or an epileptic state is reflected by ISI distributions of the involved neurons [47]. As another example, ISI distributions of otherwise similar neurons can change systematically with the anatomical location, such as within the cochlear nucleus [48], or within the somatosensory cortex [49]. While one usually models the ISI distribution as a Gamma distribution [43, 50], more complex constructions are often warranted [51, 52]. In such cases, having a model—such as ours—that can describe ISI distributions for simple as well as complex neurons is crucial for the assessment of the relationship between neuronal biophysics and the ISI distributions in a systematic way.

To make our analysis more concrete, we focus on building models describing the ISI distribution of a certain type of neurons, called Purkinje cells (PCs). These cerebellar neurons form conditional associations, and they are among the most morphologically complex neurons in mammalian brains. They have a highly elaborate dendritic arbor that forms a nearly 2-dimensional layer, which receives inputs from hundreds of thousands of other neurons. This input can lead to simple spikes (SS) or complex spikes (CS). The former are conventional action potentials fired at high frequencies in the range of 50Hz. They are caused by the input from parallel fibers, which are the axons of granule cells. The latter, on the other hand, are highly stereotyped bursts of decrementing spikes that occur in response to synaptic input from the climbing fibers. They are typically fired at a much lower frequency, in the range of a few Hz. CSs are driven in part by the large voltage-gated calcium conductance in the dendrites of Purkinje neurons [53]. Both CSs and SSs have been modeled as renewal processes. For example, an early study investigated their ISIs as a superposition of Poisson processes, where each process is attributed to a ‘firing zone’ that corresponds to a limited area of the dendritic arbor [54]. More complex mixtures of Gamma processes, two each for CSs and SSs were also explored [55]. The model family we introduce here is more general and contains both of these stochastic models as special cases, allowing us to tune the complexity of the models systematically. We will show, in particular, that a mixture of 5 or 6 Gamma distributions (many more than just two) are needed to accurately described the experimental ISI distributions of PCs of a Rhesus monkey. At the same time, we will show even the most detailed computational model of the same cells can be described by just 4 terms in the mixture, hinting at a room for improvement of biophysical models.

We conclude this article with a discussion of other applications where our method may be useful.

## Results

### The model family

**Fig 1:**
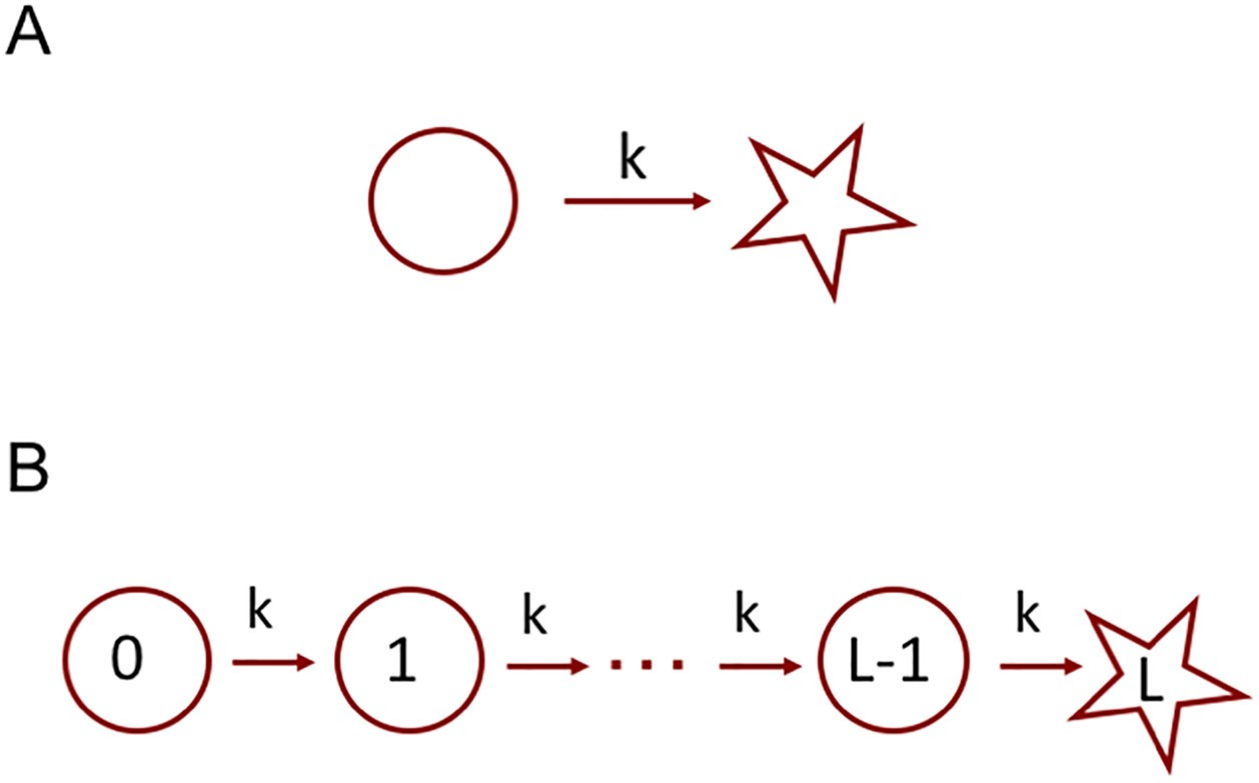
Simple FP processes. A: Exponential completion, with *k* = 1/*τ*. B: Multi-step completion, with the Erlang-distributed completion time.

**Fig 2:**
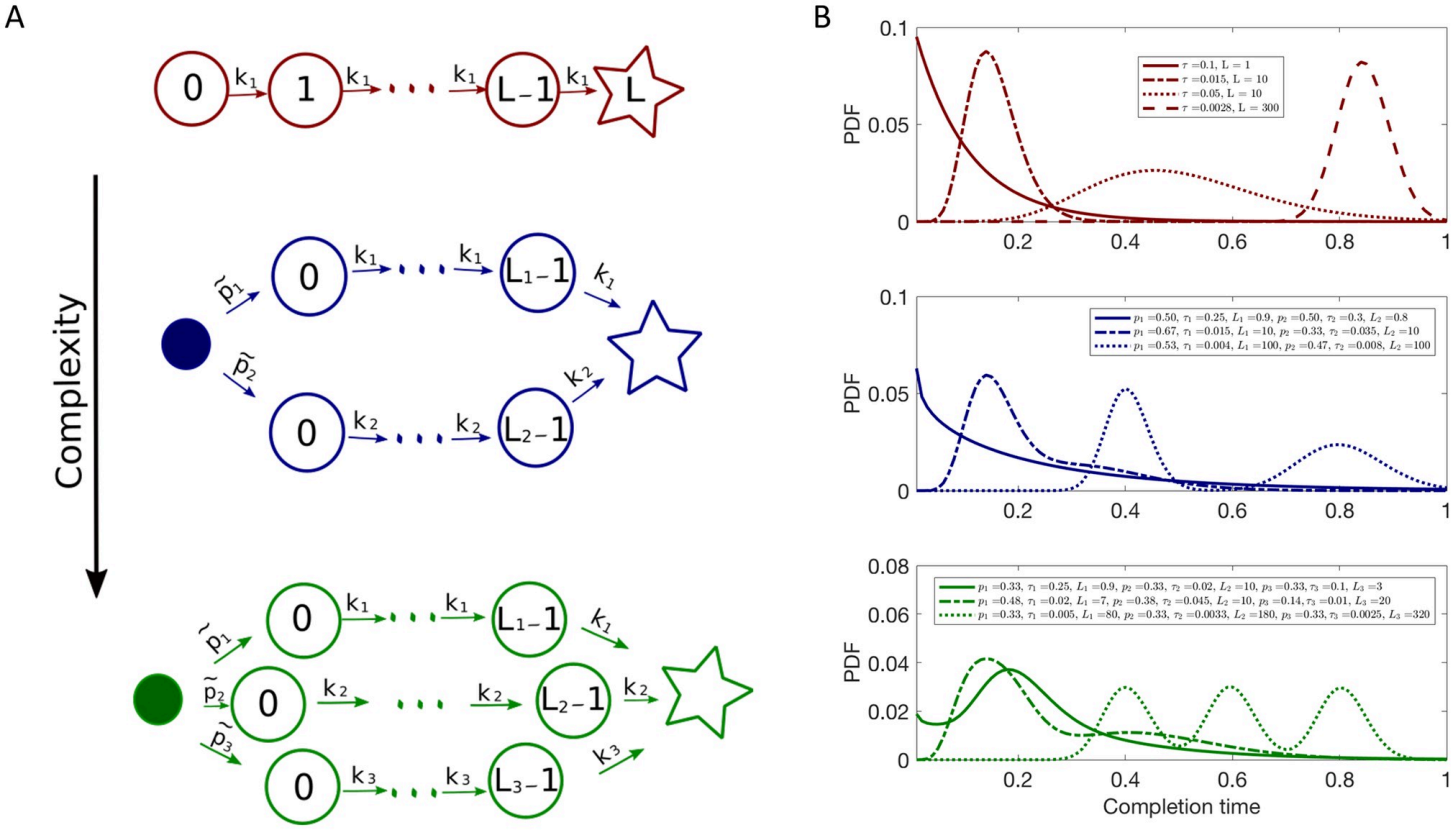
Hierarchical set of models. A: Kinetic schemes of the first three models in the hierarchical set. Each next model in the hierarchy is built by adding another completion path, where *k*_*i*_ = 1/*τ*_*i*_ is the transition rate between intermediate states, and *p*_*i*_ is the probability of completion through the path *i*. B: Examples of FP probability densities that can be generated with the corresponding models with different parameter values.

The simplest possible stochastic model to represent a FP process is a two state system as shown in Fig 1A. With a constant transition time *τ* between the initial and the absorbing state, we get an exponentially decaying completion time probability distribution *P*(*t*) = exp(−*t*/*τ*)/*τ*. A natural extension is a multi-step activation process, where the system irreversibly passes through a number of intermediate states before reaching the absorbing state, see Fig 1B. A simple induction shows that the completion probability distribution in this case is given by the Erlang distribution, Eq (1):

$$\begin{array}{c} P\left(t|\tau ,L\right)=\frac{t^{L-1}}{\tau^{L}\left(L-1\right)!}\exp\left(-t/\tau \right), \end{array}$$ (1)

where *L* corresponds to the number of intermediate states before FP and *τ* is the average transition time between the intermediate states, which we take to be the same for all states for simplicity and, as we show later, without the loss of generality. Notice that when *L* is a positive real number, Eq (1) becomes a Gamma distribution. This simple model is commonly used to describe neural ISI distributions. However, often times neural spikes exhibit more complex ISI distributions [56–61]. Motivated by these empirical findings, we built a set of models that are hierarchically organized, so that their complexity can be adapted to the quality and the quantity of empirical data by adding additional Gamma-distributed completion paths as schematically shown in Fig 2A.

The mathematical expression of our model with *M* different completion paths is:

$$\begin{array}{ll} P(t|\vec{\theta },M)= & p_{1}P(t|\tau_{1},L_{1})+p_{2}P(t|\tau_{2},L_{2})+\ldots +p_{M}P(t|\tau_{M},L_{M}),\\ p_{1}= & \frac{1}{1+x_{2}+\ldots +x_{M}},\;p_{2}=\frac{x_{2}}{1+x_{2}+\ldots +x_{M}},\;\ldots \;,\\ p_{M}= & \frac{x_{M}}{1+x_{2}+\ldots +x_{M}}, \end{array}$$ (2)

where $\vec{\theta }=\left(\tau_{1},L_{1};x_{2},\tau_{2},L_{2};\ldots ;x_{M},\tau_{M},L_{M}\right)$ are parameters to be fitted and *P*(*t*|*τ*_*i*_, *L*_*i*_) are defined as in Eq (1). Notice that when there is only one completion path, *M* = 1, with only one non-absorbing state *L*_1_ = 1, we recover the exponential distribution function with the decay time *τ*_1_. Fig 2B shows examples of FP time distributions that can emerge from models with different small values of *M* by changing parameter values. These distributions can approximate processes, such as neuronal bursts, which have multiple characteristic time scales.

We will call the union of all models $P(t|\vec{\theta },M)$, with *M* = 1, …, ∞, the *multi-path model family* of FP distributions. We will focus on Bayesian inference of phenomenological models of FP processes within this family for the rest of this work. One would like such statistical inference to be *consistent*, so that, in the limit of infinite data, one would recover the true model if it belongs to the model family being used in the inference. For an infinite model family to allow such consistent statistical inference using Bayesian approaches, it is sufficient for the family to be *nested* and *complete* [62]. Nestedness (or hierarchy) means that models within the family can be ordered in such a way that the set of solutions of a given model is contained in the set of solutions of the next model in the hierarchy. Completeness means that every data set can be fitted arbitrarily well by some (possibly very complex) model in the hierarchy.

The multi-path model family is trivially nested: if we set *p*_*M*_ = 0, then the model with *M* paths reduces to the one with *M* − 1. The proof of completeness is a bit more subtle, see Materials and methods. With that, we know that estimating the posterior probability of the model within the family given the observed data *D*, and then choosing *M* that maximizes the posterior probability *P*(*M* ∣ *D*), will typically result in consistent inference and in “selection” of the most probable model. Specifically, we need to evaluate the following integral

$$\begin{array}{c} P\left(M|D\right)\propto \int P\left(D|\vec{\theta },M\right)P\left(\vec{\theta }|M\right)d\vec{\theta }, \end{array}$$ (3)

where

$$\begin{array}{c} P\left(D|\vec{\theta },M\right)=\prod_{i}P_{M}\left(t_{i}|\vec{\theta }\right), \end{array}$$ (4)

and *t*_*i*_ is the *i*’th completion time in the experimental data set being fitted. Eq (3) comes from Eq (9) in Materials and methods, were we assumed that prior probabilities of each model in the family are the same, *P*(*M*) = const. This makes the prior unnormalized if we allow arbitrary *M*. This is not a serious practical complication. One way to interpret this is to say that, for the data sets of realistic sizes, we do not expect to explore *M* > 10 or so. This is especially true since we seek phenomenological, interpretable models, while interpreting models with a dozen of paths would be complicated, necessitating other modeling approaches. Then our method is equivalent to having a uniform prior over *M* with a finite and not too large support, where the detailed upper cutoff on the model complexity does not matter, in practice.

Unfortunately, as *M* grows in Eq (3), the involved integral becomes high-dimensional, and it is very difficult to estimate reliably. One usually assumes that the integrand is strongly peaked near the maximum likelihood value ${\vec{\theta }}_{0}$, which maximizes $P(D|\vec{\theta },M)$. A variety of approximate methods exist for the evaluation [28, 29, 63–66], which make different assumptions about the structure of the integrand near its maximum likelihood argument ${\vec{\theta }}_{0}$. We observed that, for most data sets we tried, $P(D|\vec{\theta },M)$ were far from Gaussian, thus prohibiting the use of the simple Laplace approximation to evaluate the integral [28, 63]. Therefore, we used importance sampling [64, 67] to evaluate Eq (3), see Materials and methods.

Experimental data is usually quantized in units of the experimental time resolution. To fit such data we, therefore, transform Eq (2) into its discrete time version by integrating FP probabilities over a time discretization window Δ*t*. That is, Eq (2) becomes

$$\begin{array}{cc} P_{\Delta t}\left(t|\vec{\theta },M\right)= & p_{1}\int_{t-\Delta t}^{t}P\left(t|\tau_{1},L_{1}\right)dt\\ & +p_{2}\int_{t-\Delta t}^{t}P\left(t|\tau_{2},L_{2}\right)dt+\cdots +p_{M}\int_{t-\Delta t}^{t}P\left(t|\tau_{M},L_{M}\right)dt\\ & \approx p_{1}P\left(t|\tau_{1},L_{1}\right)\Delta t+p_{2}P\left(t|\tau_{2},L_{2}\right)\Delta t+\cdots +p_{M}P\left(t|\tau_{M},L_{M}\right)\Delta t. \end{array}$$ (5)

The code to implement the multi-path model family for FPP is available at https://github.com/criver9/Inferring-FPP.git. Data generated to implement this method can be found at https://figshare.com/articles/dataset/Inter-spike_intervals_of_Purkinje_Cells/13489629.

### Model for interspike intervals for Purkinje cells

Purkinje Cells (PCs) are neurons present in the cerebellum of vertebrate animals, which participate in learning. They have large and intricate dendritic arbors and produce complex action potentials with a multiscale distribution of the interspike intervals (ISIs). Due to the complexity of the cells, their typical models involve many dozens of compartments, each described by a handful of biophysical parameters [68–72]. Crucially, the process of generating a spike can be seen as a FP process, where the neuron goes through a set of different effective states, not necessarily in a simple sequence, before crossing a certain voltage threshold (the absorbing state that results in a spike generation). Thus here we ask whether the ISI distribution for PCs, indeed, requires so many features to model well, or if, in contrast, the structural complexity of PCs does not result in a similarly high complexity of the spike generation. To answer this, we use ISIs of PCs corresponding to simple spikes of a Rhesus monkey (*Macaca mulatta*), obtained from [61], and we search for the best phenomenological model of this distribution using our approach.

**Fig 3:**
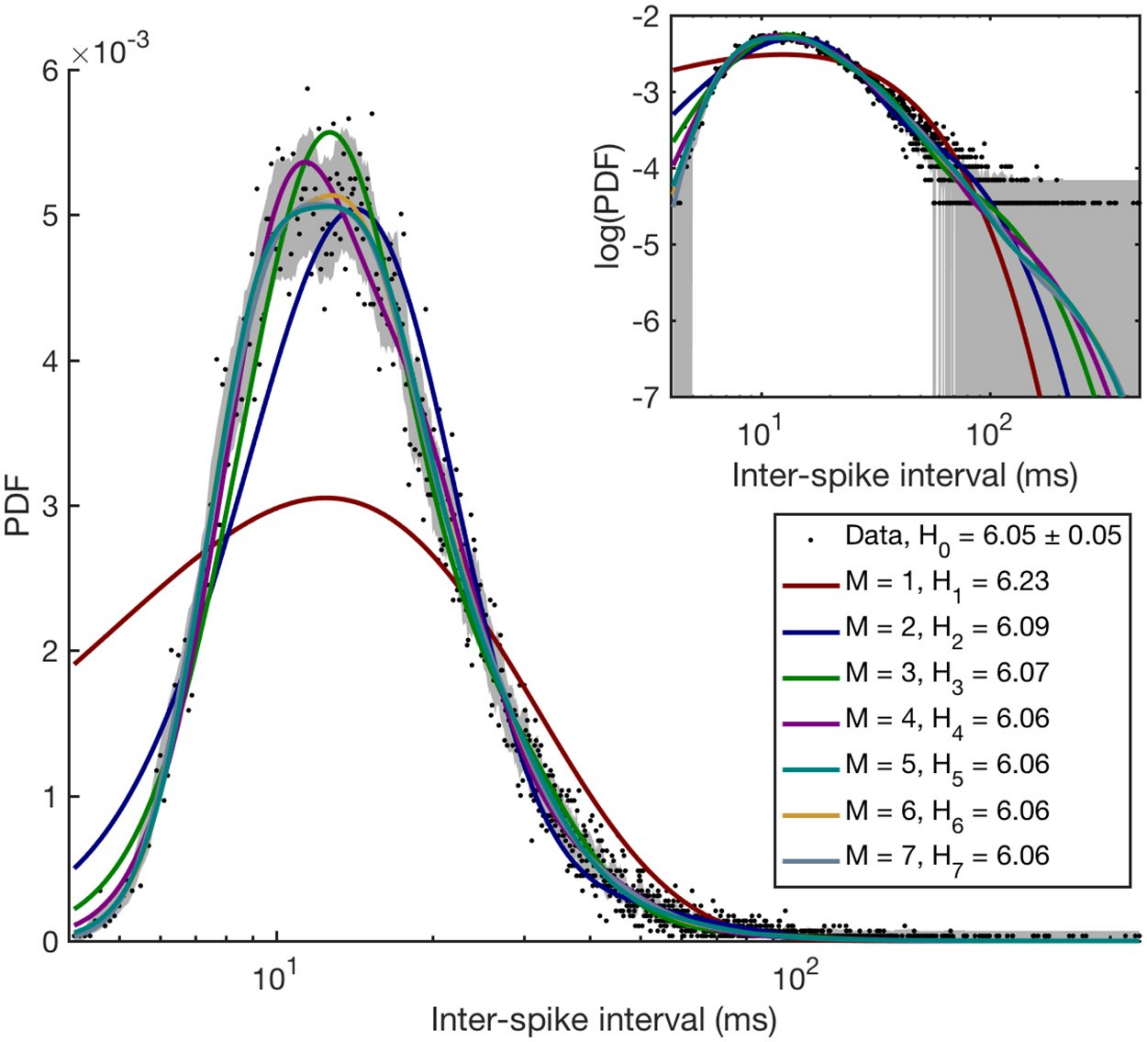
Best fit models, *M* = 1…7, for Purkinje cells ISI distribution. Dots indicate the histogram of the real data, and the grey band denotes the standard error of every dot. Color lines show the average fit line sampled from the posterior distribution of each of the first seven models in the hierarchy, error bands (too narrow to see) on these fits where estimated using the standard deviation from the sampled curves (see Materials and methods). The legend illustrates how the cross-entropy between the data distribution and the model fits decreases with the model complexity towards the entropy of the data distribution itself. Note that the horizontal axis is logarithmic. Inset: same data, but on log-log axes.

Fig 3 shows the best fits for each of the model in our hierarchy, *M* ≤ 7, to the PC ISI distribution data. The figure and Table 1 suggest that the simplest phenomenological model of the process contains about *M* = 5 effective independent paths (for this data set, we cannot discriminate between models with *M* = 5, 6 based on the values of *P*(*D* ∣ *M*)). Notice that, by gradually adding additional completion paths, we can approximate not only the right tail of the distribution, but also the left tail—the behavior at early times. We measure this quality of fit by showing, in Fig 3, the entropy of the probability distribution, $H_{0}=-\sum_{i=1}^{N}p_{i}\ln p_{i}$ (evaluated using the Bayesian entropy estimator [73]), of the data being fitted, as well as the cross-entropy, $H_{M}=-\sum_{i=1}^{N}p_{i}\ln P_{\Delta t}\left(t_{i}|\vec{\theta },M\right)$, between the data and each of the best fit models with different *M* (this corresponds to minus the normalized value of the log-likelihood, Eq (4)). To the extent that *H*_*M*_ approaches *H*_0_ for larger *M*, the fits are quite good. And since *H*_*M*_ ≈ *H*_*M*+ 1_ for large *M*, the fits stop becoming much better, so that the Bayesian Model Selection [33] then penalizes models with large *M*. The model with *M* ≈ 5 turns out to have the highest marginal likelihood, though the models with *M* = 6, 7 are close.

**Table 1:** Model selection results for ISI probability distribution of experimental PC. Natural logarithm of the marginal likelihoods of the first seven models in the hierarchy are shown for *N* = 28966 spikes (the full data set). Numbers following the ± sign are the standard deviation of the marginal likelihood, estimated using importance sampling (see Materials and methods). Since we show log likelihoods instead of likelihoods, the standard deviations transform into asymmetric errors around the mean, and both asymmetric errors are shown. The model with the highest marginal likelihood, *M* = 5, is highlighted. Note that the model with *M* = 6 cannot be ruled out, as it has very similar marginal likelihood.

*M*	ln*P*(*D* ∣ *M*)
1	−180379.2 +/- (0.1/0.1)
2	−176368.516 +/- (0.001/0.001)
3	−175826.323 +/- (0.008/0.008)
4	−175694.65 +/- (0.02/0.02)
5	−175649.89 +/- (0.08/0.08)
6	−175651.7 +/- (0.3/0.5)
7	−175655.5 +/- (0.6/1.5)

We next check how the selected model depends on the amount of data being fitted. As seen in Table 2, increasing the number of spikes in the data set from 1000 to ∼30000 allows us to identify finer details in the data which require more accurate models to be fitted. Thus the most likely model has *M* = 2 for a small data set, gradually increasing to *M* = 5 for full data. Since the last three-fold increase in the amount of data does not result in a further growth of the best *M*, we conclude that the phenomenological model likely has reached the complexity needed to explain the system, and the model with *M* = 5 is, in some sense, equivalent to the full complexity of simple spike generation of a real Purkinje cell.

**Table 2:** Model selection as a function of the number of samples. First row shows the size of the data set, 1000…28966, and the rest of the table shows the logarithm of the marginal likelihood of each model in the family for these data. Error bars on the log-marginal likelihoods are in S1 Table. As the number of samples increases, more complex models are required to explain the details of PC spiking, but the complexity eventually saturates, presumably having matched the complexity of the real cells observed at the given experimental accuracy.

ln*P*(*D* ∣ *M*)						
*M*	1000	5000	10000	15000	20000	28966
1	-6192	-30901	-61775	-92835	-124017	-180379
2	**-6133**	-30427	-60673	-91189	-121736	-176368
3	-6134	-30369	-60536	-90915	-121356	-175826
4	-6143	**-30349**	-60487	-90859	-121262	-175694
5	-6155	-30354	**-60482**	**-90842**	**-121230**	**-175649**
6	———	-30362	-60491	-90850	-121236	-175651

This analysis illustrates two crucial points. First, a relatively simple model with *M* ≈ 5 is able to explain the experimental ISI distribution from a complex neuron, so that much of the physiological complexity of the cell does not translate into a functional complexity, at least at the scale of a simple spike generation. Second, quantitatively fitting the data favors models with *M* ≥ 5 by a factor of ∼ 10^20^. Indeed, from Table 1, we see that the difference of log-likelihoods of models with *M* = 5 and *M* = 4 is ≈ 17695 − 17650 = 45, which translates into the ratio of likelihoods of ≈ *e*^45^ ≈ 3.5 ⋅ 10^19^. In other words, PC spiking is not trivially simple, and guessing this ISI model without the automated inference procedure developed here would likely be impossible.

### Model for ISI of synthetic PC

One of our interests is to develop phenomenological models that are able to predict the change in the FP distributions for a system under the influence of various external perturbations. We would like to illustrate this using PCs. However, we are not aware of readily available large, precise data sets measuring the ISI distribution in PCs under external perturbations. Thus instead we focus on synthetic data, generated using a biophysically realistic, multi-compartmental model that resembles the morphologically complex structure of PCs, the Miyasho et al. model [69], which is a modified version of the earlier De Schutter and Bower model [68]. To illustrate the complexity of the Miyasho model, we point out that it uses 1087 compartments to describe the dendritic arbor of a PC and one compartment for the soma. Additionally, the dynamics are defined by around 150 parameters that specify 12 different types of voltage-gated ion channels [69].

**Fig 4:**
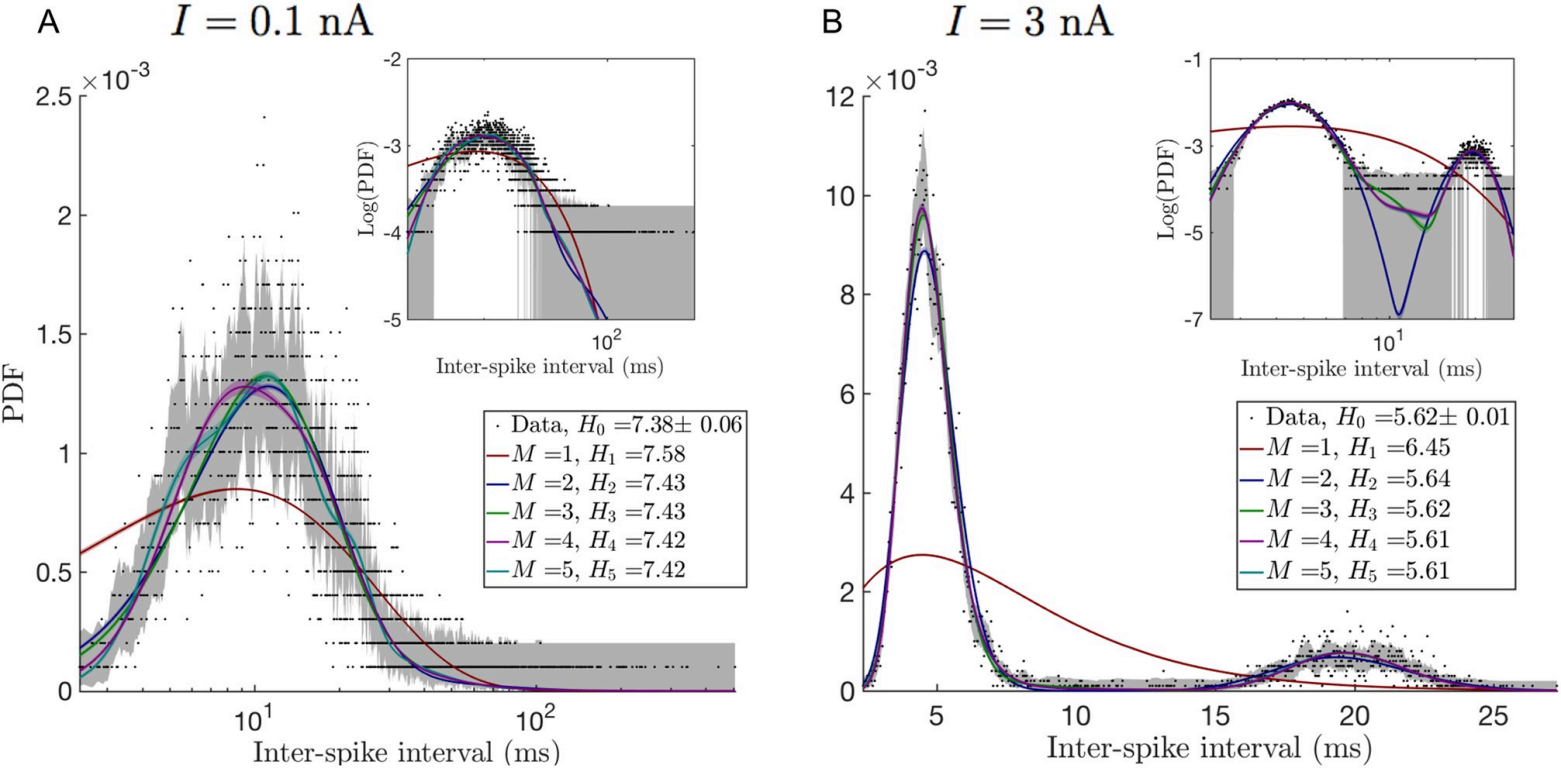
Best fits for different models in the model family for the distribution of ISIs of synthetic PCs. Color lines and color bands show the mean and standard deviation respectively of different models sampled from the posterior distribution of each of the first five models in the family (see details in Materials and methods). The legend shows how the cross entropy decreases with the model complexity towards its minimum value of the entropy of the histogram of the observed data. According to Table 3, 4 paths are needed to explain the ISI characteristics of synthetic PCs under different external conditions. A: injected current *I* = 0.1 nA, and B: *I* = 3 nA. Insets in both panels show the same data, but on log-log axes.

We used this model to simulate the behavior of the membrane potential dynamics of a PC, affected by different electric currents injected into the soma. White noise currents with standard deviation *σ* = 3 nA and mean values *I* = 0.1, 0.5, 0.7, 1, 2, 3 nA where injected, thus generating six different data sets, with which to explore the ISI probability distributions of the PC model. Following the procedure described earlier, we selected the simplest phenomenological model that can explain the ISI statistics of the PC model, but in this case we focus on optimizing the marginal likelihood over all stimulus values simultaneously. Fig 4 shows the best model fits for two different injected currents which produce qualitatively different ISI distributions. Fits for other current values can be found in S1 Fig. To build the optimal model for all injected currents simultaneously, we estimate the marginal likelihood of each model in the family for *M* ≤ 5 for each of the synthetic data sets, see Table 3. Since for different currents, the ISI generated are independent of each other, the log-likelihood for the entire data set is simply the sum of log-likelihoods for each *I*. As always, we choose the optimal model as the one with the largest overall log-likelihood.

**Table 3:** Model selection results for ISI of synthetic PCs. Marginal likelihood of the first five models in the family for each data set, corresponding to the six different injected currents. Error bars on the log-marginal likelihoods are in S2 Table. Last column shows that a model with 4 completion paths is optimal over the combined data. Asterisk marks those cases where the optimal parameter values fell at the boundary of the search space, usually because there were paths with near-zero flux through them (see Materials and methods). Note that the numbers in the first two columns increase monotonically with *M*, so that the best model in the family is not found for *M* ≤ 5. We chose to truncate the exploration at *M* = 5 since we are interested in the overall maximum of the log-likelihood for all *I*, which is reached at *M* = 4 (last column).

ln *P*(*D*∣*M*)							
*M*	*I* = 0.1 nA	*I* = 0.5 nA	*I* = 0.7 nA	*I* = 1.0 nA	*I* = 2.0 nA	*I* = 3.0 nA	Total
1	-75436	-71282	-66821	-66309	-64654	-64488	-408992
2	-74070	-70034	-65283	-63578	-58932	-56462	-388359
3	-74019	-70001	**-65238**	-63520	**-58773**	**-56211**	-387762
4	-74003	-69990	-65239	**-63518**	-58794	-56213	**-387757**
5	-73994	-69976	-65251*	-63530*	-58806*	-56226*	-387784

**Fig 5:**
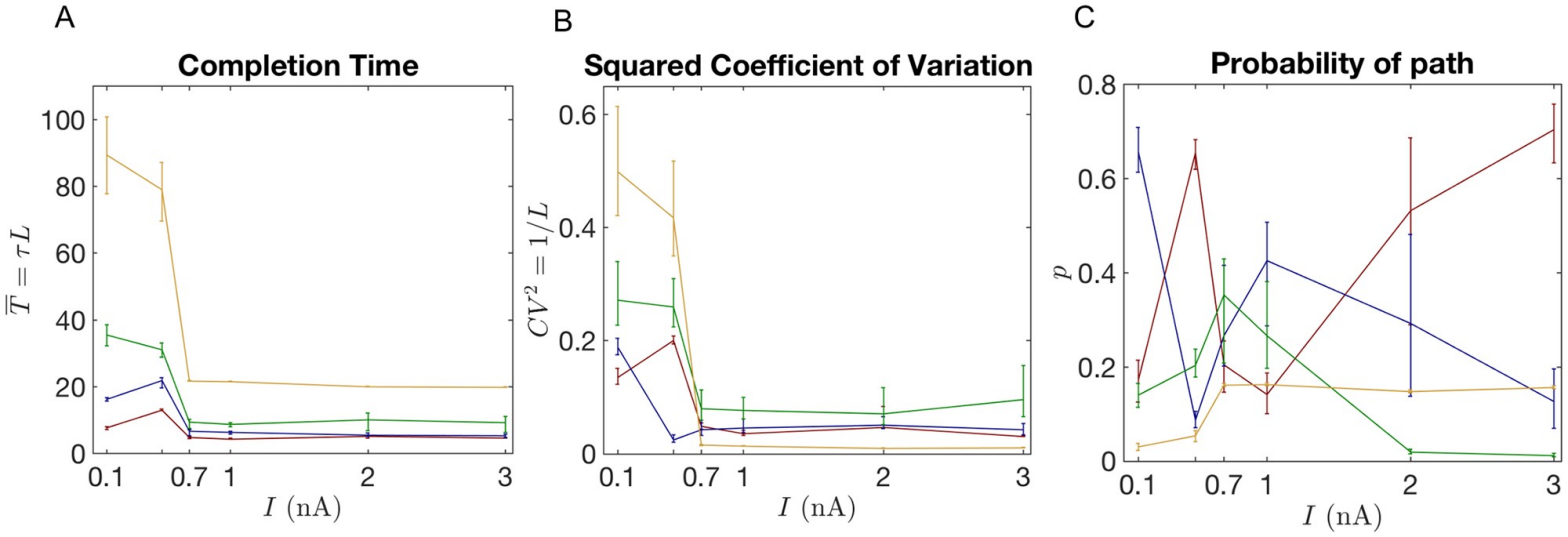
Properties of completion paths change as a function of the external parameter for the best model selected across all experiments. A: Average completion times for each of the *M* = 4 independent paths are plotted as a function of the injected current in the soma, *I*. Color (same in (B) and (C)) identifies paths according to how long they take to complete the process on average. B: Coefficient of variation and C: probability of taking each of the independent paths of the model as a function of *I*.

Table 3 shows that, for our data sets, *M* = 4 effective independent paths are enough to explain simultaneously the PCs behavior under six different injection currents. As can be seen in S2 Fig, when the injected current increases the cell goes from the non-bursting to the bursting state, and the entropy of the completion time distribution decreases (see Fig 4 and S1 Fig). Table 3 indicates that higher entropy distributions, corresponding to *I* = 0.1, 0.5 nA need *M* ≥ 5 completion paths to be properly explained. Lower entropy distributions, on the other hand, not only require fewer paths, but also more deterministic paths, as can be observed from the coefficient of variation estimates in Fig 5. This suggests, that under low external stimulus (*I* < 0.5 nA), spike generation in the cell can happen through multiple pathways. Instead, when a certain current threshold is reached (*I* > 0.5 nA), only a few of these pathways get activated. Nonetheless, more than one pathway is needed even for high currents, since, at least, two time scales are required to explain the bursting activity.

In Fig 5, we explore how the properties of the model selected in Table 3 (*M* = 4) change as a function of the injected current, *I*. Each independent path is described by specifying its average completion time ${\bar{T}}_{i}=\tau_{i}L_{i}$, the coefficient of variation ${\text{CV}}_{i}^{2}=1/L_{i}$, and the probability *p*_*i*_ of completion along this path, and these three quantities are plotted for each path for different values of *I*. There is a sharp change in these features when the PC transitions from a non-bursting to a bursting state, between *I* = 0.5 and 0.7 nA. For example, completion times and coefficient of variation for all paths drop drastically at this point. In particular, S3 Fig shows that the paths with the longest completion time explain very different aspects of the non-bursting and the bursting ISI distributions. For the non-bursting cases, these paths help to fit mostly the tails. Instead, for the bursting cases, these paths explain the intra-burst time interval, which happens to be a much more deterministic process, as can be seen from the behavior of the coefficients of variation, Fig 5.

To test whether the phenomenological model correctly captures the time scales of the underlying biophysical processes, we predict the ISI distribution for input currents that the model was not exposed to during fitting. To achieve this we first need to determine a relationship between model parameters and the input current means, which we can then use to infer model parameters for currents different from the ones used for fitting the model. As our test case, we employed the model with *M* = 4 and tracked the dependence of its parameters on the current as shown in Fig 5. A priori it is unclear how to build correspondence between the four model paths for separate input currents. In our example in Fig 5, we chose to establish the correspondence by ordering the paths according to their completion time, thus relating the model paths with the smallest completion time, then the second to smallest and so on. This ordering provides relationships between input currents and all model parameters, based on which we can infer parameter values for new current values using linear interpolation (for currents that fall between two fitted values) or linear extrapolation (for currents outside of the fitted range). We note that the choice to relate parameter values by completion time rather than another parameter is arbitrary. Indeed there are many possibilities to create the pathway correspondence for different current values. Besides ordering based on average completion time (confront Fig 5) we also tested ordering based on the coefficient of variation or the probability path which led to no improvement over the presented case (not shown). While it is possible that other orderings can lead to better predictions we leave a more systematic exploration of this aspect for future work.

**Fig 6:**
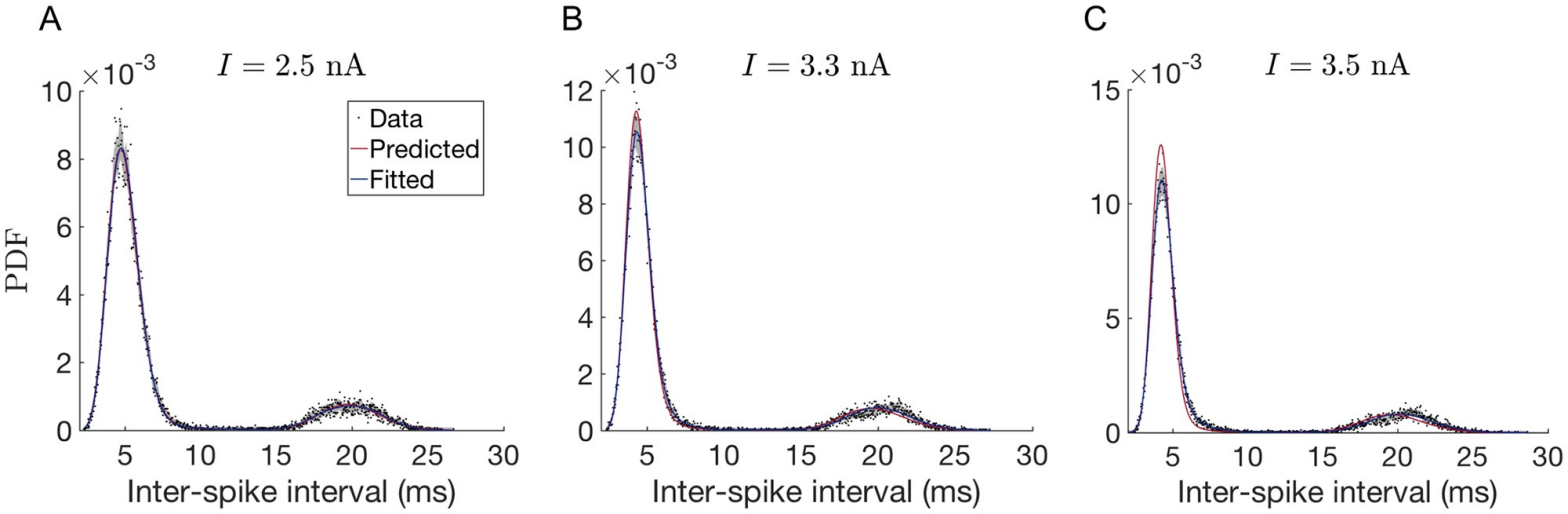
Predicted PDFs for non-measured values of the injected current. Predicted model (in red) was obtained by interpolating parameter values from Fig 5. It is compared with the model (in blue) fitted directly to data. (A) Prediction for *I* = 2.5 nA (interpolation). (B) and (C) Prediction for *I* = 3.3 and *I* = 3.5 nA respectively (extrapolation).

To validate our predictions, we generated new data for mean currents *I* = 2.5, 3.3, and 3.5 nA and compared predicted ISI distributions to the simulation results (see Fig 6). The predicted model for *I* = 2.5 nA was obtained by linearly interpolating the statistical properties shown in Fig 5 between the known values at *I* = 2.0 and 3.0 nA. Then we used the following relations to infer the parameters of the model: $L_{i}=1/{\text{CV}}_{i}^{2}$, $\tau_{i}={\bar{T}}_{i}/L_{i}$ and *x*_*i*_ = *p*_*i*_/*p*_1_. Fig 6A shows that the predicted model is almost indistinguishable from the fitted one. Similarly, the predicted model for *I* = 3.3 and *I* = 3.5 nA, is obtained by linearly extrapolating the statistical properties from the last two known values at *I* = 2.0 and 3.0 nA. These showed very good agreement with the respective simulated data (Fig 6B and 6C).

**Fig 7:**
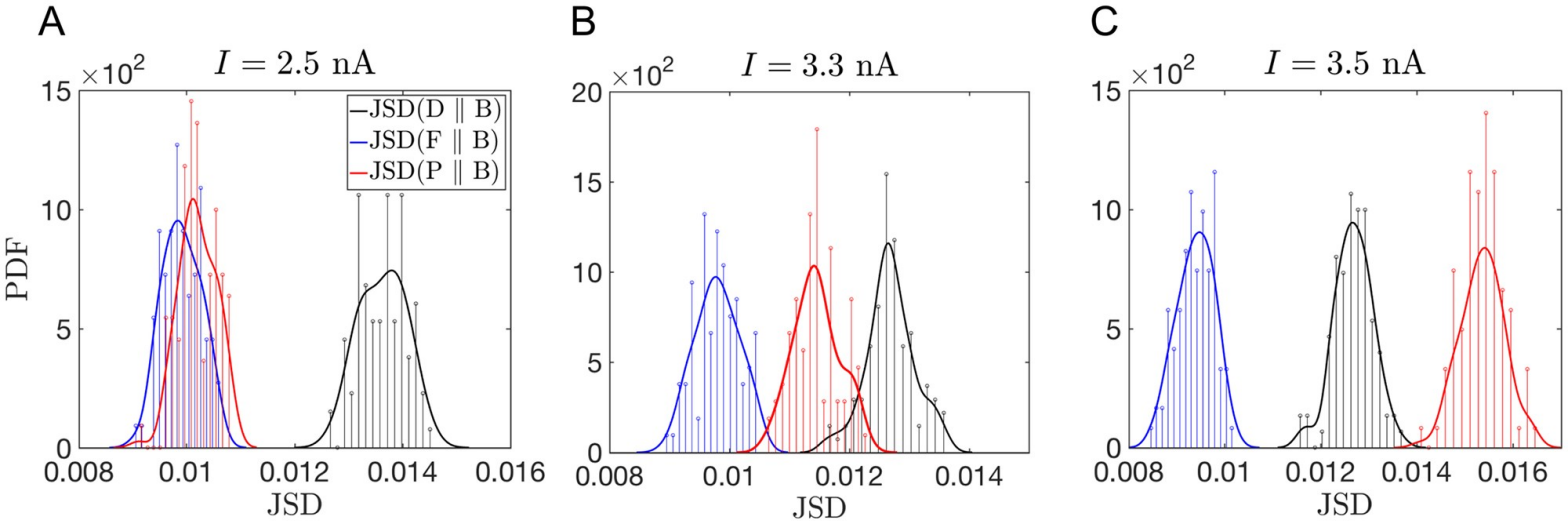
Quantifying quality of the predictions. We plot the histograms of the JSD between the test data set and the bootstrapped samples from the validation data set (in black), the JSD between the bootstrapped validation data sets and models fitted to each of these data sets (in blue), and the JSD between the bootstrapped data and the prediction based on interpolating or extrapolating the model parameters fitted to the original data (in red). To the extent that the distributions are close, predictions are good. A-C: Data for *I* = 2.7, 3.3, 3.5 nA, respectively. The first is interpolation, the other two are extrapolations.

To quantify the accuracy of these predictions, we need to calculate their quality with respect to some baseline. We chose the Jensen-Shannon Divergence (JSD) [74] as a measure of the quality of fit, and we measure it relative to two baselines. First, we quantify how an extrapolated or an interpolated prediction compares to the fit done directly on a data set; certainly the fit is expected to outperform the prediction. Second, we check how two statistically equivalent realizations of data fit each other; this should be the ceiling, which neither the fit nor the prediction can outperform (if both are not overfitted). Both of these baselines depend on the specific data set used, and thus one needs to estimate probability distributions of the relevant JSDs, rather than their single values. However, generating data from the PC model takes hours even on a modern computer, and hence we generate only a single additional, validation, data set beyond the training and the testing sets, which we then additionally bootstrap (resample with replacement) to produce statistics of the JSDs. Specifically, Fig 7 plot histograms of (i) the JSD between the test data and the bootstrapped versions of the validation data (this is the statistics that requires us to have two independent samples, test and validation, to remain unbiased), (ii) the JSD between the bootstrapped validation data and fits to these data, and (iii) the JSD between the prediction and the bootstrapped validation data. Our first observation is that all three JSD distributions are very close to each other, indicating very good fits and predictions. For *I* = 2.5 nA, the fits/predictions have smaller JSD than different realizations of data have with themselves, which is consistent a very good fit, and suggests, as expected, that the variability across bootstrapped data sets is somewhat larger than would have been across independent samples. As *I* increases, and interpolation gives way to extrapolation, the prediction quality deteriorates (still remaining only a few percent worse than the fits).

### Inferring mechanistic constraints

Our approach to modeling FP time probability distribution is purely phenomenological. However, the multi-path model family allows us additionally to constrain mechanistic, biophysical models of the underlying processes. Specifically, we can make predictions for the minimal number of intermediate states that a mechanistic model requires to explain the data. Indeed, for any FP problem, the short-time behavior of the completion probability density provides information about the length of the shortest completion path [34, 75]. That is, assume that the process starts in a state *i* and ends at the absorbing state *j* of an arbitrary Markovian chemical reaction network. Then, at short times, the completion probability density can be approximated as *ρ*_*ij*_ ∝ *t*^*m*^, where *m* is the number of intermediate states of the shortest path connecting states *i* and *j* [75]. In principle, this means that by estimating the exponent of the power law that fits the left tail of the completion time distribution, one can put a lower limit on the number of intermediate states in a mechanistic model. Then any candidate model with a fewer number of steps can be rejected.

In practice, making use of this result is hard because it requires data with very high temporal resolution, and a very well sampled left tail. However, our multi-path representation allows for an extension of the approach to the case where the sampling is good, but the time resolution may not be sufficient for simpler methods. Once the most probable model in the model family is selected and fitted, we propose to determine if the first few fastest events can be explained by a single independent path *i* of length *L*_*i*_. We use 50 events in our analysis, which provides for a sufficient number of the events to seek a power law fit, and yet is small enough so that only the very end of the left tail is explored. Since at short time scales the Cumulative Distribution Function (CDF) of the FP time probability density is $\propto t^{L_{i}}$ (from Eq (1)), one can insist that any mechanistic model built to describe the data will need at least *L*_*i*_ states, establishing a lower bound on the size of the network.

**Fig 8:**
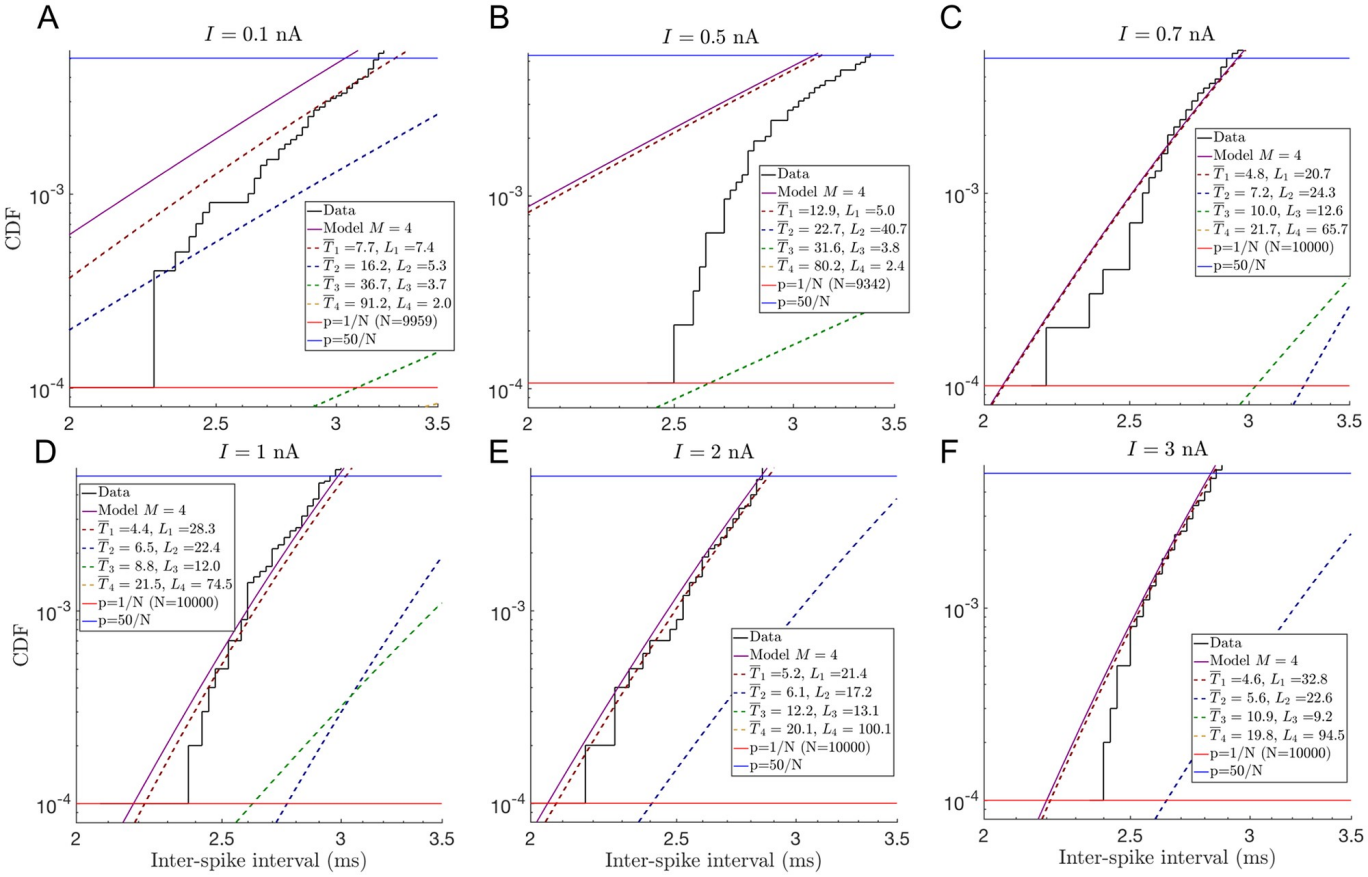
Decomposition of the Cumulative Distribution Functions (CDFs) of completion time at early times into the four completion pathways. Black line represents the CDFs from data; horizontal red and blue lines in each plot correspond to the probability of the 1st and the 50th events, respectively. Solid purple lines are CDFs from the best-fitted model with *M* = 4, and each of the dashed lines represents contributions from the constituent completion paths. A-F: *I* = 0.1, 0.5, 0.7, 1.0, 2.0, 3.0 nA, respectively. Panels C-F show that the first fifty events can be explained by one or more paths with ∼20…30 intermediate states. Therefore, any biophysically accurate reaction network explaining these data needs to have at least >20 internal states. Notice that, even though a model with *M* = 4 is optimal over all values of *I* according to Table 3, it does not explain the early time behavior in panels (A,B).

For concreteness, the short time behaviors of the CDFs obtained from the best model, *M* = 4, describing the ISIs of PCs under six different injected currents are shown in Fig 8. Only for *I* = 0.7 nA the first 50 events (0.5% of sample size) can be explained by a single path with ∼20 intermediate states, while for larger values of *I*, the distribution can be fitted by one or more of such paths. In all of these cases, it is thus clear that any realistic biophysical model of a PC must include, at least, ∼20 internal states.

## Discussion

In this study, we developed a mathematical structure (multi-path model family) to infer phenomenological models describing FP time distributions for biological processes. As an example of application of our approach, we show that this representation allows us to build models capable of describing the complexity of the ISI distributions of PCs by successfully explaining not only the bulk, but also the tails of the distribution. Our results show that the process of a spike generation in PCs is more complex than a simple renewal process with a Gamma-distributed completion time, which is typically used in the field. For simple spikes, *M* ≥ 5 independent Gamma-distributed paths are required. We also showed that only *M* ≈ 4 paths (11 independent parameters) are needed to explain the behavior of synthetic PCs over all injected current values *I* > 0.5 nA. This illustrates that (i) morphological complexity of PCs notwithstanding, their dynamics is not very complex at the level of the FP time distribution, and (ii) our fully phenomenological approach can, nonetheless, point out when biophysically-realistic models are inconsistent with features of experimental data. By identifying how parameters of the inferred model change with the external stimulus and extrapolating or interpolating them, we can predict the FP time distribution of the system in response to novel stimulus values. These predictions focus not just on the mean and the variance, but on the entire completion time distribution, and we have shown that the predictions are remarkably accurate, as compared to statistical fluctuations in the data themselves. Finally, we showed how our purely phenomenological approach can establish the minimum size of a mechanistically accurate biochemical network underlying the system, at least for well-sampled data sets.

Intriguingly, our data driven approach, which suggests 4-6 gamma processes to model PC spiking, produces models that are more complex than the hand-crafted model by Shin et al., which suggests 2 Gamma processes for simple spikes [55]. Thus we suggest that our modeling approach can be used to validate mathematical models by investigating relationships between the time-scales of neuronal spiking and detailed biophysical properties of PCs, such as particular types of ion channels, or morphological properties. Future studies could use a morphologically complex model, such as [69] which we used here, to investigate time-scales by altering specific properties and re-fitting our phenomenological model family. The resulting model complexities for the altered and original mechanistic model can be compared to assess the influence of particular biophysical and morphological properties on the time-scales of spike trains.

Ours is certainly not the first attempt at reconstructing kinetic diagrams of a process using FP data [76, 77]. However, previous approaches have focused on mechanistic models and did not consider general complete and nested model families, unlike our proposed multi-path model family. This family has additional useful properties: (i) Models in this family result in FP distributions that are positive and normalized, unlike polynomial or Fourier expansions of the distribution, or various simple moment closure techniques. (ii) This family results in true FP distributions—that is, distributions that have support only on positive real values of the completion time. (iii) Some very complex FP processes, with multiple steps within a completion path, are described by simple models in our hierarchy. The last observation, we think, is the most important feature that allowed our model family to successfully model the spike generation of highly complex neurons, the Purkinje cells. While the multi-path family certainly works well, it is only one of many possible hierarchies that satisfies nestedness and completeness properties, and hence can be used within our framework. Different hierarchies may be better suited for phenomenological modeling of different biological processes [27], and the quality of fit within a family may reveal which family better matches salient properties of the modeled processes. We hope to develop such additional model families and explore their pros and cons in subsequent papers.

Within our model, we assumed that every completion time is independent and identically distributed. This is a strong assumption, which is not necessarily realized. For example, serial correlations of ISIs violate this assumption. Early studies found no serial correlation in ISIs of PCs [54], however, more recently it was suggested that, while complex spikes do not show serial correlations, simple spikes do [55]. Additionally, if each spike within a complex spike, i. e., a burst, is treated like any other spike, successive ISIs further exhibit a dependence (i.e., within a burst, a short ISI is usually followed by another short ISI). In the future, it should be possible to extend our approach to model such processes by either modeling the statistics of FP time for a sequence of events, or by extending the model family to incorporate a latent variable that controls the dependence among subsequent completion events.

Our models offer only limited understanding of the mechanistic details of the modeled biological process. Nonetheless, there are many advantages to our approach, and phenomenological modeling, in general. Indeed, the complexity that biological processes have acquired over eons of evolution oftentimes makes building detailed microscopic models an extremely challenging task. And yet the functional properties of the behavior might be rather simple, with the structural complexity existing, for example, to ensure robustness of the function to various perturbations. Then focusing on the phenomenological model allows us to elucidate, predict, and eventually use properties of the functional behavior even if microscopic details of the mechanisms used to produce it remain unclear. Our specific approach to phenomenological modeling is different from many others in that it does not coarse-grain a microscopic model (requiring a laborious task of building one as an intermediate step), but rather it refines phenomenological models, adding progressively more details until the functional behavior is well approximated. Bayesian model selection is used to find the optimal point in the refinement hierarchy. The computational advantages of taking such an adaptive, refining approach can be huge, especially when the studied complex system exhibits a simple behavior. The computational complexity of our approach is dominated by searching for optimal fits, which scales linearly with the data set size, and exponentially with the model complexity. However, the latter is rarely more than a few dozen parameters even for very complex systems, such as the PCs, at least for realistic experimental resolution and data set sizes. Thus we expect our approach to be useful for modeling any biological system for which (i) the quantity that we need to predict is the completion time, (ii) the underlying biophysics is very complex, with microscopic details not always affecting the macroscopic completion properties, and where (iii) large, high quality experimental data sets are available for different experimental conditions, requiring (iv) to predict the behavior of the system as a function of these conditions, for their yet-untested values.

## Materials and methods

### Completeness

Here we show that the model family studied in this work, Eq (5), is complete. That is, any data set describing the distribution of the completion times of the first passage process can be approximated arbitrarily well by a gamma mixture model with sufficient complexity.

We note that experimentally measured and numerically simulated completion times are constrained by finite resolutions which essentially discretizes the time axis. Thus we can write the completion time likelihood as a multinomial

$$\begin{array}{c} L\left(\vec{q}|\vec{n}\right)=q_{1}^{n_{1}}q_{2}^{n_{2}}\ldots {\left(1-q_{1}-q_{2}-\ldots q_{K-1}\right)}^{\left(N-n_{1}-n_{2}-\ldots n_{K-1}\right)}, \end{array}$$ (6)

where *n*_*i*_ counts how often the completion time falls into the *i*th out of *K* time interval bins (*t*_*i*_ − Δ*t*, *t*_*i*_], *N* is the total number of completion time events, and *q*_*i*_ is the probability of completion in the time interval defined by bin *i*, given by $q_{i}=P_{\Delta t}\left(t_{i}|\vec{\theta },M\right)$ (see Eq (5)). Trivially, the maximum of $L(\vec{q}|\vec{n})$ is achieved when *q*_1_ = *n*_1_/*N*, *q*_2_ = *n*_2_/*N*, …*q*_*K*_ = *n*_*K*_/*N*. Therefore, our aim must be to construct a model that can bring $\vec{q}$ arbitrarily close to this maximum. The rational of the proof is to have a path per time bin whose average waiting time is the center of the respective time bin and whose variance can get arbitrarily small, effectively approximating a delta function. That is, we want to construct a model such that for any *ϵ* > 0, we have $n_{i}/N-∊\leq P_{\Delta t}\left(t_{i}|\vec{\theta },K\right)\leq n_{i}/N+∊$.

To prove this we set the parameters in Eq (5) to what follows. For the probability of every gamma path take *p*_*i*_ = *n*_*i*_/*N*, with expected completion time given by *T*_*i*_ = *L*_*i*_ *τ*_*i*_ = *t*_*i*_ − Δ*t*/2 and variance (arbitrarily small) $\sigma_{i}^{2}=T_{i}\tau_{i}=\frac{\Delta t^{2}}{4}{∊}_{i}$, where ${∊}_{i}=\text{min}\left(\frac{∊}{p_{1}+p_{2}+\ldots +p_{i-1}+p_{i+1}+\ldots p_{K}},\frac{∊}{p_{i}}\right)$. Then, we can show that:

$$\begin{array}{cc} P_{\Delta t}\left(t_{i}|\vec{\theta },K\right) & =\frac{n_{1}}{N}\int_{t_{i}-\Delta t}^{t_{i}}P\left(\tau_{1},L_{1}\right)dt+\frac{n_{2}}{N}\int_{t_{i}-\Delta t}^{t_{i}}P\left(\tau_{2},L_{2}\right)dt+\ldots \\ & \;+\frac{n_{K}}{N}\int_{t_{i}-\Delta t}^{t_{i}}P\left(\tau_{k},L_{K}\right)dt\\ & \leq \epsilon_{i}\left[\frac{n_{1}}{N}+\ldots \frac{n_{i-1}}{N}+\frac{n_{i+1}}{N}+\ldots \frac{n_{K}}{N}\right]+\frac{n_{i}}{N}\\ & \leq \epsilon +\frac{n_{i}}{N} \end{array}$$ (7)

where we used Chebyshev’s inequality ($\text{Pr}(|t-T_{i}\left|\geq \alpha \sigma_{i}\right)\leq \frac{1}{\alpha^{2}}$, with $\alpha =1/\sqrt{{∊}_{i}}$) to set a bound to all the integrals but the *i*th. For the *i*th integral we note that, since most of the probability mass falls in this bin, it reaches close to one and is naturally bounded by one. This concludes the upper bound on the *q*_*i*_. For the lower bound we simply subtract one from both sides of the Chebyshev inequality and multiply by negative one to get $\text{Pr}(|t-T_{i}\left|\leq \alpha \sigma_{i}\right)\geq 1-\frac{1}{\alpha^{2}}$. This gives a bound for the *i*th integral of Eq (7):

$$P_{\Delta t}\left(t_{i}|\vec{\theta },K\right)\geq \frac{n_{i}}{N}\int_{t_{i}-\Delta t}^{t_{i}}P\left(t|\tau_{i},L_{i}\right)dt\geq \frac{n_{i}}{N}\left(1-\epsilon_{i}\right)\geq \frac{n_{i}}{N}-\epsilon ,$$ (8)

showing that this model family can approximate any sufficiently smooth distribution arbitrarily well. In real applications, we may not need to have as many paths as there are bins to achieve a high approximation accuracy, so the construction above is the worst case scenario.

### Model selection

To choose the most likely model from the family, we evaluate and maximize the marginal probability of each model *M*:

$$\begin{array}{c} P\left(M|D\right)=\frac{P(D|M)P(M)}{P(D)}\propto P\left(D|M\right), \end{array}$$ (9)

where we assumed that all models in the hierarchy are *a priori* equally likely. The likelihood *P*(*D* ∣ *M*) is given by:

$$\begin{array}{c} P\left(D|M\right)=\int P\left(D|\vec{\theta },M\right)P\left(\vec{\theta }|M\right)d\vec{\theta }, \end{array}$$ (10)

where the likelihood of the data set and the prior are chosen to be:

$$\begin{array}{c} P(D|\vec{\theta },M)=\prod_{i=1}^{K}P_{\Delta t}{\left(t_{i}|\vec{\theta },M\right)}^{n_{i}}, \end{array}$$ (11)

$$\begin{array}{c} P(\vec{\theta }|M)=\frac{1}{{\left(Z_{x}\right)}^{M-1}}\prod_{j=1}^{M}\frac{\exp(-\frac{\tau_{j}}{Z_{\tau }})}{Z_{\tau }}\frac{\exp(-\frac{L_{j}}{Z_{L}})}{Z_{L}}. \end{array}$$ (12)

Here $P_{\Delta t}\left(t|\vec{\theta },M\right)$ is given by Eq (5), and *n*_*i*_ is the number of events with completion time between (*t*_*i*_ − Δ*t*, *t*_*i*_). The priors over *L*_*j*_ and *τ*_*j*_ are chosen to be exponential, ∼exp(−*L*_*j*_/*Z*_*L*_) and ∼exp(−*τ*_*j*_/*Z*_*τ*_), respectively. The values of *Z*_*L*_ and *Z*_*τ*_ must then be set in such a way that the priors are wide compared to the measured time scales, so that *τL* can be as small as the temporal resolution or larger than the longest completion times observed. Throughout our study we set them to *Z*_*L*_ = 20 and *Z*_*τ*_ = 20 ms. The prior over *x*_*j*_ is chosen to be uniform between 0 and *Z*_*x*_, which was set to *Z*_*x*_ = 10^3^. This allows each path to be sufficiently dominant over the others so that even distinguishing the existence of other paths given the data set sizes we explore is hard. In the limit of a large number of paths, priors over multinomial distributions *p*_*j*_ are known to concentrate probabilities in just a few of the possible outcomes, depending on the properties of the prior used [73]. This could be problematic, and may be addressed by using a Dirichlet prior on *p*_*j*_ (instead of the uniform prior on *x*_*j*_) and then choosing the hyperparameters of the prior with Bayesian model selection as well [73]. However, since we never considered more than 7 paths, the effects of the priors are not dramatic, and our choice is easier computationally. Finally, we note that our choice of the priors means that the parameters are *a priori* uncorrelated among themselves.

In most cases, the integration in Eq (10) is analytically intractable. A typical approach in such a case is to use the Laplace approximation to compute the integral. However, in our problems, the posterior distributions fall much slower than Gaussians, ruining the quality of the Laplace approximation. Thus we used importance sampling [67, 78] instead. Specifically, we sampled from the multi-variate normal distribution $G\left(\vec{\theta }\right)=\det{\left(2\pi \Sigma \right)}^{-\frac{1}{2}}\exp\left(-\frac{1}{2}{\left(\vec{\theta }-{\vec{\theta }}^{*}\right)}^{\prime }\Sigma^{-1}\left(\vec{\theta }-{\vec{\theta }}^{*}\right)\right)$ centered at the optimal value ${\vec{\theta }}^{*}$ of the integrand $F\left(\vec{\theta }\right)≔P\left(D|\vec{\theta },M\right)P\left(\vec{\theta }|M\right)$ with the covariance matrix Σ is defined by the Hessian of $F(\vec{\theta })$:

$$\begin{array}{c} {\left(\Sigma^{-1}\right)}_{ij}={\left(-\mathrm{Hess}\log F{|}_{{\vec{\theta }}^{*}}\right)}_{ij}\equiv -\frac{\partial^{2}\log F}{\partial \theta_{i}\partial \theta_{j}}{|}_{{\vec{\theta }}^{*}}. \end{array}$$ (13)

This way we ensured that $G(\vec{\theta })>0$ for $F(\vec{\theta })>0$, at least around the domain of the local optimum at ${\vec{\theta }}^{*}$. See below for details of how we estimated the covariance matrices. Then the importance sampling estimate of the integral in Eq (10) is

$$\begin{array}{c} P\left(D|M\right)∼\frac{1}{N}\sum_{i=1}^{N}\frac{P\left(D|{\vec{\theta }}_{i},M\right)P\left({\vec{\theta }}_{i}|M\right)}{G({\vec{\theta }}_{i})}, \end{array}$$ (14)

where ${\vec{\theta }}_{i}∼N\left({\vec{\theta }}^{*},\Sigma \right)$ and we used *N* = 10^5^ samples to achieve the desired accuracy. Since the likelihood values exceeded numerical resolution, we instead computed the ln *P*(*D* ∣ *M*):

$$\begin{array}{c} \ln P\left(D|M\right)∼\ln F\left({\vec{\theta }}^{*}\right)+\ln(\sum_{i=1}^{N}\frac{\exp(\log F\left({\vec{\theta }}_{i}\right)-\log F\left({\vec{\theta }}^{*}\right))}{G({\vec{\theta }}_{i})})-\ln N. \end{array}$$ (15)

Furthermore, the associated variance of the estimation of the marginal likelihood can be estimated by reusing the same samples as follows:

$$\begin{array}{cc} \text{Var}(P(D|M)) & ∼\frac{1}{N^{2}}\text{Var}(\sum_{i=1}^{N}\frac{F({\vec{\theta }}_{i})}{G({\vec{\theta }}_{i})})\\ & ∼\frac{1}{N}(\frac{1}{N}\sum_{i=1}^{N}{\left(\frac{F({\vec{\theta }}_{i})}{G({\vec{\theta }}_{i})}\right)}^{2}-{\left(\frac{1}{N}\sum_{i=1}^{N}\frac{F({\vec{\theta }}_{i})}{G({\vec{\theta }}_{i})}\right)}^{2}). \end{array}$$ (16)

Because the variance is small, as seen in Table 1 and S1 and S2 Tables, we conclude that the importance sampling has converged [78].

#### Covariance matrix estimation

Application of our importance sampling scheme requires knowing the maximum of the integrand and the Hessian around the optimum. The optimal values ${\vec{\theta }}^{*}$ were obtained using the MATLAB function fminsearchbnd. We used MATLAB version R2017a for our analysis. Most of the optimal values obtained for different models and data sets fell in the interior of the parameter’s domain; we mark those where the optimal values fell at the boundary with an asterisk everywhere in the text.

We first explain how we computed the covariance matrix for the cases where the optimal values fell in the interior of the parameters’ domain set. Using Eq (11) to estimate the Hessian, we get

$$\begin{array}{cc} -\frac{\partial^{2}\log F}{\partial \theta_{k}\partial \theta_{j}}{|}_{{\vec{\theta }}^{*}} & =-\sum_{i}^{M}n_{i}\frac{\partial^{2}\log\left(P_{\Delta t}\left(t_{i}|\vec{\theta },M\right)\right)}{\partial \theta_{k}\partial \theta_{j}}{|}_{{\vec{\theta }}^{*}}-\frac{\partial^{2}\log P\left(\vec{\theta }|M\right)}{\partial \theta_{k}\partial \theta_{j}}{|}_{{\vec{\theta }}^{*}}\\ & =\sum_{i}^{M}n_{i}{\left[\frac{1}{P_{\Delta t}{\left(t_{i}|\vec{\theta },M\right)}^{2}}\frac{\partial P_{\Delta t}\left(t_{i}|\vec{\theta },M\right)}{\partial \theta_{k}}\frac{\partial P_{\Delta t}\left(t_{i}|\vec{\theta },K\right)}{\partial \theta_{j}}\right|}_{{\vec{\theta }}^{*}}\\ & -\frac{1}{P_{\Delta t}\left(t_{i}|\vec{\theta },M\right)}\frac{\partial^{2}P_{\Delta t}\left(t_{i}|\vec{\theta },M\right)}{\partial \theta_{k}\partial \theta_{j}}{|}_{{\vec{\theta }}^{*}}]. \end{array}$$ (17)

Notice that the contribution to the Hessian coming from the prior in the previous expression cancels out. We then evaluated Eq 17 numerically using Eq (5).

For those cases, for which the optimal values are located at the boundary of the parameters’ domain due to the presence of a trivial completion path we use the following trick. Given that the flux through a certain path *j* is zero, the likelihood *P*(*D*∣*θ*, *M*) stays constant for all values of *τ*_*j*_ and *L*_*j*_ corresponding to this trivial path. However, the prior decays exponentially and therefore $F(\vec{\theta })$ also decays exponentially in the directions of *τ*_*j*_ and *L*_*j*_. The optimal value of *F*(*θ*) can be written as $\left({\vec{x}}_{p},x_{d}=0,\tau_{d}=0,L_{d}=0\right)$ with ${\vec{x}}_{p}$ is the best fit for the previous model in the family, with only *d* − 1 completion paths. Then the covariance matrix is:

$$\begin{array}{c} \Sigma =(\begin{array}{cc} \begin{array}{c} \Sigma_{p} \end{array} & \text{0}\\ \text{0} & \begin{array}{ccc} \alpha_{x}^{2} & 0 & 0\\ 0 & \alpha_{\tau }^{2} & 0\\ 0 & 0 & \alpha_{L}^{2} \end{array} \end{array}), \end{array}$$ (18)

where Σ_*p*_ is the covariance matrix at the best fit of the previous model in the family; $\alpha_{x}^{2}$ is an upper bound on the variance along the parameter controlling the probability flux through *d*-th completion path estimated from the symmetric function *F*_*s*_(*θ*) = *F*(|*θ*|). We used *α*_*x*_ = 0.01 for all the cases marked with an asterisk in Table 3. On the other hand *α*_*τ*_ and *α*_*L*_ where estimated using the variances of the independent exponential distributions of the prior, Eq (12), *Z*_*τ*_ = *Z*_*L*_ = 20. We chose $\alpha_{\tau }^{2}=\alpha_{L}^{2}={\left(3\sigma_{\tau }\right)}^{2}=3600$. Notice that, along these last two directions where *F*(*θ*) decays exponentially we chose the variance of the importance distribution nine times larger in these two directions to make sure that it contains most of the important domain of *F*(*θ*).

#### Parameter degeneracy

The likelihoods that we obtain often have multiple modes that correspond to parameter degeneracy, which arises by relabeling the completion paths. To account for this degeneracy in calculating the integral to estimate the marginal likelihood, we multiplied the likelihoods of each model with *M* gamma pathways by (*M* − 1)!. Here we use *M* − 1 instead of *M* because the first path is different from the others: transition rate to this path is set to one and is used as a reference.

#### Generalized Bayesian model selection

In order to find the model in the family that best fits the simultaneous description of the system under *s* different external conditions, we need to estimate the integral Eq (10) for *s* independent data sets,

$$\begin{array}{cc} P(D_{1},D_{2},\ldots ,D_{s}|M) & =\int P\left(D_{1},D_{2},\ldots ,D_{s}|{\vec{\theta }}_{1},{\vec{\theta }}_{2},\ldots ,{\vec{\theta }}_{s},M\right)P\left({\vec{\theta }}_{1},{\vec{\theta }}_{2},\ldots ,{\vec{\theta }}_{s}|M\right)d\vec{\theta }\\ & =\prod_{j=1}^{s}\int P\left(D_{j}|{\vec{\theta }}_{j},M\right)P\left({\vec{\theta }}_{j}|M\right)d\vec{\theta_{j}}. \end{array}$$ (19)

The last equality results from each data set having its own, independent set of parameters. Taking the natural logarithm on both sides of Eq (19), we obtain the following result, which we used to compute the values in Table 3:

$$\begin{array}{c} \ln P\left(D_{1},D_{2},\ldots ,D_{s}|M\right)=\sum_{j=1}^{s}\ln P\left(D_{j}|M\right) \end{array}$$ (20)

Note that Bayesian model selection helps us resolve many complications often present in fitting models like ours. For example, one may imagine having multiple pathways with similar parameter values, which would require very large data sets to lift the degeneracy. In such a situation, our Bayesian model selection procedure would keep just one pathway instead of two, until there is sufficient data to explore the more complicated model.

### Expected values and uncertainty of fits

The fits and the error bars for curves for all of the fitted models in all Figures are the expected values and the standard deviations of the model curves over the posterior probability distributions. That is,

$$\begin{array}{ccc} \left\langle f(t|M)\right\rangle & = & \int f\left(t|\vec{\theta },M\right)P\left(\vec{\theta }|D,M\right)d\vec{\theta }, \end{array}$$ (21)

$$\begin{array}{ccc} \mathrm{Var}(f(t|M)) & = & \int {\left(f\left(t|\vec{\theta },M\right)-\langle f(t|M)\rangle \right)}^{2}P\left(\vec{\theta }|D,M\right)d\vec{\theta }, \end{array}$$ (22)

where $f\left(t|\vec{\theta },M\right)=P_{\Delta t}\left(t|\vec{\theta },M\right)$, and the posterior probability is

$$\begin{array}{c} P\left(\vec{\theta }|D,M\right)=\frac{P\left(D|\vec{\theta },M\right)P\left(\vec{\theta }|M\right)}{P(D|M)}=\frac{F(\vec{\theta })}{P(D|M)}. \end{array}$$ (23)

As explained above, we used importance sampling to estimate the expectation values. For example, notice that Eq (21) can be rewritten as

$$\begin{array}{c} \left\langle f(t|M)\right\rangle =\frac{\int f\left(t|\vec{\theta },M\right)F\left(\vec{\theta }\right)d\vec{\theta }}{\int F\left(\vec{\theta }\right)d\vec{\theta }}. \end{array}$$ (24)

Using Eq (15), this becomes

$$\begin{array}{c} \langle \hat{f}\left(t|M\right)\rangle \approx \frac{\sum_{i=1}^{N}\frac{f\left(t|{\vec{\theta }}_{i},M\right)\exp\left(\log F\left({\vec{\theta }}_{i}\right)-\log F\left({\vec{\theta }}^{*}\right)\right)}{G({\vec{\theta }}_{i})}}{\sum_{i=1}^{N}\frac{\exp(\log F\left({\vec{\theta }}_{i}\right)-\log F\left({\vec{\theta }}^{*}\right))}{G({\vec{\theta }}_{i})}} \end{array}$$ (25)

Similarly, for the variance, we have

$$\begin{array}{c} \mathrm{Var}\left(f\left(t|M\right)\right)=\frac{\int {\left(f\left(t|\vec{\theta },M\right)-\langle f(t|M)\rangle \right)}^{2}F\left(\vec{\theta }\right)d\vec{\theta }}{\int F\left(\vec{\theta }\right)d\vec{\theta }}, \end{array}$$ (26)

which results in

$$\begin{array}{c} \mathrm{Var}\left(\hat{f}\left(t|M\right)\right)\approx \frac{\sum_{i=1}^{N}\frac{f{\left(t|{\vec{\theta }}_{i},M\right)}^{2}\exp\left(\log F\left({\vec{\theta }}_{i}\right)-\log F\left({\vec{\theta }}^{*}\right)\right)}{G({\vec{\theta }}_{i})}}{\sum_{i=1}^{N}\frac{\exp(\log F\left({\vec{\theta }}_{i}\right)-\log F\left({\vec{\theta }}^{*}\right))}{G({\vec{\theta }}_{i})}}-{\left\langle \hat{f}\left(t|M\right)\right\rangle }^{2}. \end{array}$$ (27)

## Supporting information

S1 Fig Best fits for different models in the family to the experimental Purkinje cells interspike interval data. Color lines and bands (the latter often too narrow to be seen) show the mean and the standard deviation of different models sampled from the posterior distribution of each of the first five models in the family. The legends illustrate the decrease of the cross entropy with the model complexity towards its minimum value of the entropy of the histogram of the observed data. According to Table 3, 4 paths are needed to explain the ISI characteristics of synthetic under different external conditions. (A, B, C, D) injected currents *I* = 0.5, 0.7, 1.0, 2.0 nA, respectively.
(TIF)

S2 Fig Simulated PC membrane potential using the multi-compartmental model proposed in [69] for A: low (*I* = 0.5 nA) and B: high (*I* = 3 nA) values of the injected current. (TIF)

S3 Fig Decomposition of the completion time PDF into contributions from different paths for (A) *I* = 0.1 nA and (B) *I* = 3.0 nA. Insets show the same data in log-log units. In (A), the two pathways with the shortest completion time explain the bulk of the distribution while the pathway with the longest average completion time approximate the right tail of the distribution. In (B), pathways with shortest/longest completion time contribute mostly to the intra/inter burst time scales.
(TIF)

S1 Table Same results as in Table 2, but with likelihood error estimate using importance sampling, see Materials and methods for details. See Table 1 for conventions used.
(PDF)

S2 Table Same results as in Table 3, but with likelihood error estimate using importance sampling, see Materials and methods for details. See Table 1 for conventions used.
(PDF)
