# Supplementary figures and images for: Inferring phenomenological models of first passage processes
Source: PLoS Comput Biol. 2021 Mar 5;17(3):e1008740. doi: 10.1371/journal.pcbi.1008740 (PMC7968746; doi:10.1371/journal.pcbi.1008740)

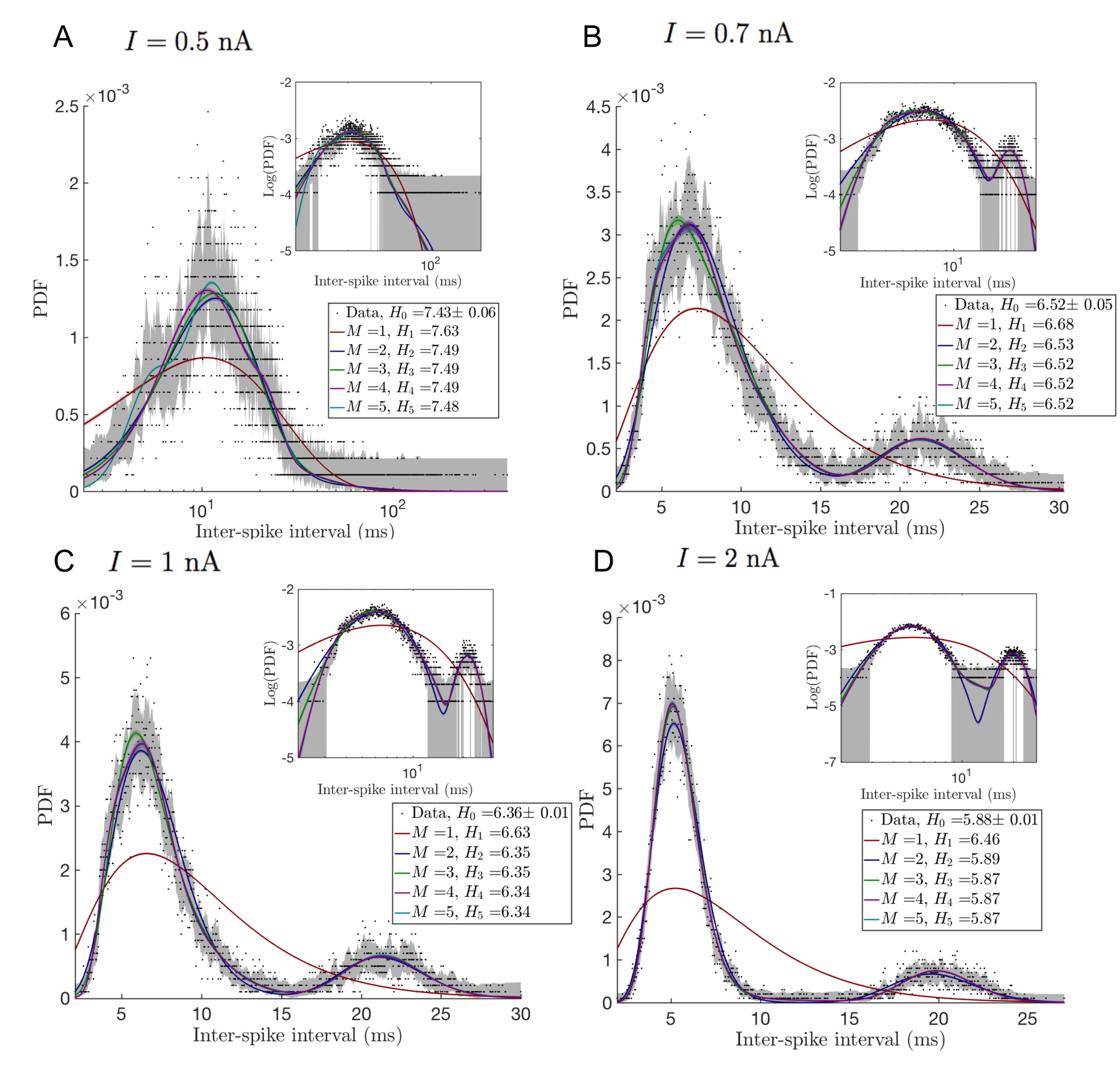

Supplement: S1 Fig — Color lines and bands (the latter often too narrow to be seen) show the mean and the standard deviation of different models sampled from the posterior distribution of each of the first five models in the family. The legends illustrate the decrease of the cross entropy with the model complexity towards its minimum value of the entropy of the histogram of the observed data. According to Table 3, 4 paths are needed to explain the ISI characteristics of synthetic under different external conditions. (A, B, C, D) injected currents I = 0.5, 0.7, 1.0, 2.0 nA, respectively. (TIF) [file pcbi.1008740.s001.tif]

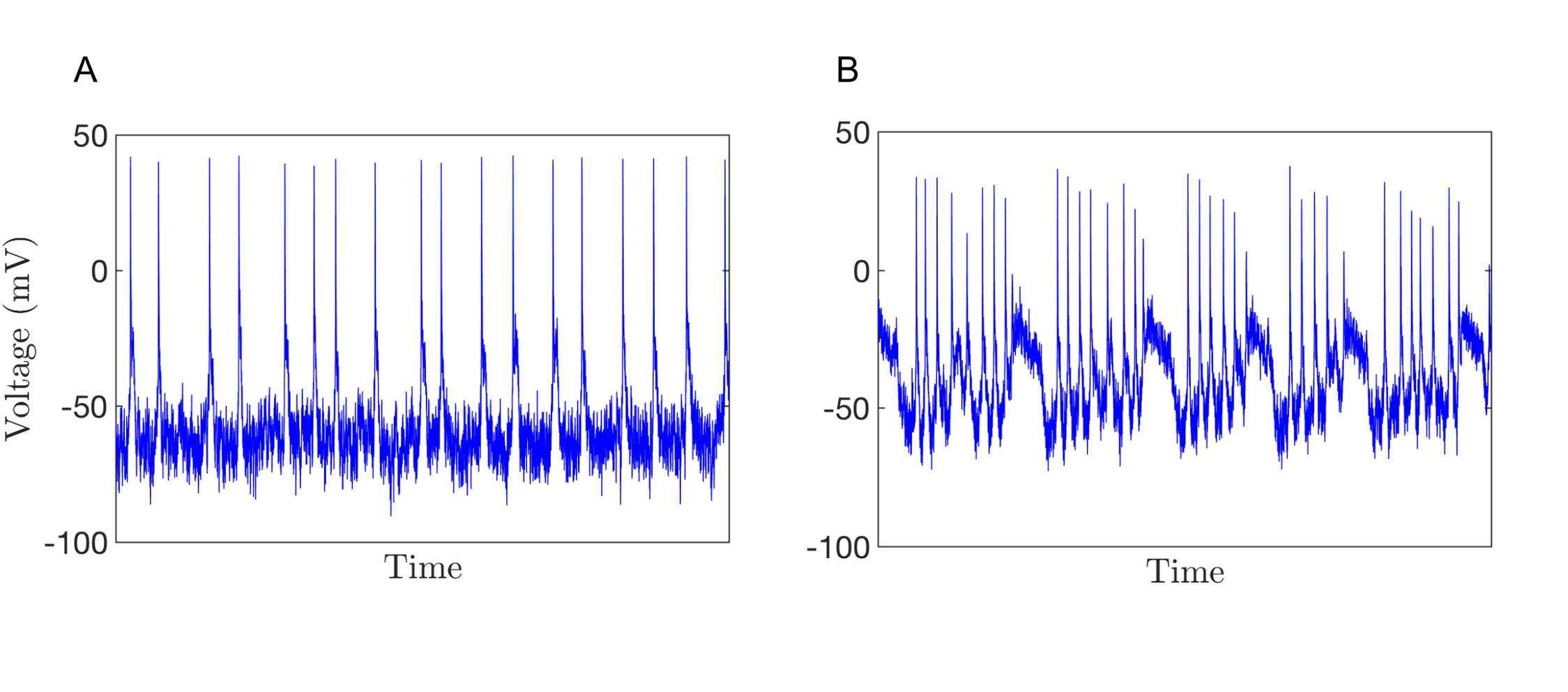

Supplement: S2 Fig — (TIF) [file pcbi.1008740.s002.tif]

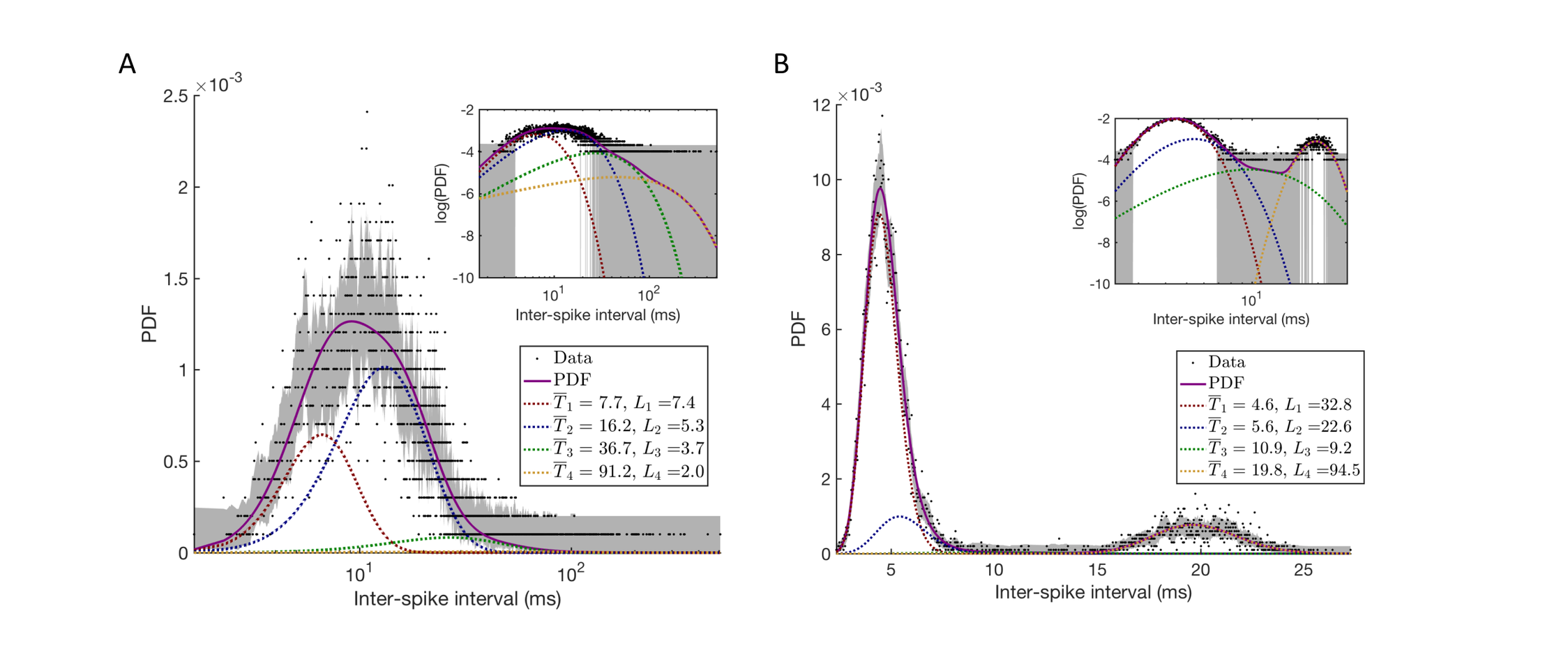

Supplement: S3 Fig — Insets show the same data in log-log units. In (A), the two pathways with the shortest completion time explain the bulk of the distribution while the pathway with the longest average completion time approximate the right tail of the distribution. In (B), pathways with shortest/longest completion time contribute mostly to the intra/inter burst time scales. (TIF) [file pcbi.1008740.s003.tif]
