# Supplementary material for: Inferring phenomenological models of first passage processes
Source: PLoS Comput Biol. 2021 Mar 5;17(3):e1008740. doi: 10.1371/journal.pcbi.1008740 (PMC7968746; doi:10.1371/journal.pcbi.1008740)
Supplement: S1 Table — See Table 1 for conventions used. (PDF) [file pcbi.1008740.s004.pdf]

$\ln P(D \mid M)$			
M	1000	5000	10000
1	-6192.088 +/- (0.001/0.001)	-30901.890 +/- (0.003/0.003)	-61775.0 +/- (0.4/0.6)
2	**-6133.63 +/- (0.02/0.02)**	-30427.107 +/- (0.003/0.003)	-60673.466 +/- (0.003/0.003)
3	-6134.30 +/- (0.04/0.04)	-30369.7 +/- (0.1/0.1)	-60536.7 +/- (0.1/0.1)
4	-6143.5 +/- (0.2/0.3)	**-30349.7 +/- (0.2/0.3)**	-60487.22 +/- (0.04/0.04)
5	-6155.7 +/- (0.2/0.3)	-30354.1 +/- (0.4/0.8)	**-60482.7 +/- (0.3/0.4)**
6	——————-	-30362.0 +/- (0.2/0.2)	-60491.1+/- (0.3/0.4)

$\ln P(D \mid M)$			
M	15000	20000	28966
1	-92835.9 +/- (0.1/0.1)	-124017.4 +/- (0.7/2)	-180379.2 +/- (0.1/0.1)
2	-91189.678 +/- (0.001/0.001)	-121736.298 +/- (0.001/0.001)	-176368.516 +/- (0.001/0.001)
3	-90915.45 +/- (0.03/0.03)	-121356.56 +/- (0.01/0.01)	-175826.323 +/- (0.008/0.008)
4	-90859.2 +/- (0.2/0.2)	-121262.88 +/- (0.04/0.04)	-175694.65 +/- (0.02/0.02)
5	**-90842.2 +/- (0.2/0.3)**	**-121230.4 +/- (0.6/1)**	**-175649.89 +/- (0.08/0.08)**
6	-90850.5 +/- (0.2/0.3)	-121236.7 +/- (0.4/0.8)	-175651.7 +/- (0.3/0.5)
