# Supplementary material for: Inferring phenomenological models of first passage processes
Source: PLoS Comput Biol. 2021 Mar 5;17(3):e1008740. doi: 10.1371/journal.pcbi.1008740 (PMC7968746; doi:10.1371/journal.pcbi.1008740)
Supplement: S2 Table — See Table 1 for conventions used. (PDF) [file pcbi.1008740.s005.pdf]

$\ln P(D \mid M)$			
M	$I = 0.1$ nA	$I = 0.5$ nA	$I = 0.7$ nA
1	−75436.4 +/- (0.4/0.9)	−71282.06 +/- (0.02/0.03)	−66820.9217 +/- (0.0002/0.0002)
2	−74070.155 +/- (0.002/0.002)	−70034.057 +/- (0.002/0.002)	−65283.280 +/- (0.008/0.008)
3	−74018.5 +/- (0.1/0.2)	−70001.3 +/- (0.1/0.1)	**−65238.09 +/- (0.04/0.04)**
4	−74003.0 +/- (0.1/0.2)	−69989.7 +/- (0.7/3)	−65239.3 +/- (0.1/0.2)
5	−73994.3 +/- (0.1/0.1)	−69976.3 +/- (0.2/0.2)	−65251.0* +/- (0.5/1)

$\ln P(D \mid M)$			
M	$I = 1.0$ nA	$I = 2.0$ nA	$I = 3.0$ nA
1	−66309.5805 +/- (0.0002/0.0002)	−64654.4834 +/- (0.0002/0.0002)	−64488.8135 +/- (0.0002/0.0002)
2	−63578.467 +/- (0.005/0.005)	−58931.665 +/- (0.007/0.007)	−56461.89 +/- (0.02/0.02)
3	−63520.01 +/- (0.03/0.03)	**−58773.2 +/- (0.2/0.2)**	**−56211.50 +/- (0.01/0.01)**
4	**−63518.13 +/- (0.07/0.08)**	−58794.51 +/- (0.06/0.06)	−56212.77 +/- (0.05/0.05)
5	−63529.7* +/- (0.3/0.5)	−58806.6* +/- (0.3/0.5)	−56226.6* +/- (0.1/0.1)

$\ln P(D \mid M)$	
M	Total
1	−408992.2 +/- (0.4/0.9)
2	−388359.51 +/- (0.02/0.02)
3	−387762.6 +/- (0.2/0.3)
4	**−387756.7 +/- (0.7/3)**
5	−387784.5 +/- (0.7/2)
